# Biphasic Glucocorticoid Rhythm in One-Month-Old Infants: Reflection of a Developing HPA-Axis?

**DOI:** 10.1210/clinem/dgz089

**Published:** 2019-10-25

**Authors:** Jonneke J Hollanders, Bibian van der Voorn, Paul de Goede, Alyssa A Toorop, Lisette R Dijkstra, Adriaan Honig, Joost Rotteveel, Koert M Dolman, Andries Kalsbeek, Martijn J J Finken

**Affiliations:** 1 Pediatric Endocrinology, Emma Children’s Hospital, Amsterdam University Medical Center, Vrije Universiteit Amsterdam, Amsterdam, The Netherlands; 2 Department of Paediatric Endocrinology, Obesity Center Centrum voor Gezond Gewicht (CGG) , Sophia Children’s Hospital, Rotterdam, The Netherlands; 3 Laboratory of Endocrinology, Amsterdam University Medical Center, University of Amsterdam, Amsterdam Gastroenterology & Metabolism, Amsterdam, The Netherlands; 4 Department of Psychiatry Obstetrics and Pediatrics (POP) , Onze Lieve Vrouwe Gasthuis (OLVG), Amsterdam, The Netherlands; 5 Netherlands Institute for Neuroscience (NIN), Royal Dutch Academy of Arts and Sciences (KNAW), Amsterdam, Netherlands

**Keywords:** circadian rhythm, human milk, cortisol, cortisone, early-life development, adrenal

## Abstract

**Context:**

The hypothalamus-pituitary-adrenal (HPA) axis displays a diurnal rhythm. However, little is known about its development in early life.

**Objective:**

To describe HPA-axis activity and study possible influencing factors in 1-month-old infants.

**Design:**

Observational.

**Setting:**

Amsterdam University Medical Center, location VU University Medical Center (VUMC), and Onze Lieve Vrouwe Gasthuis (OLVG), Amsterdam.

**Participants:**

Fifty-five mother-infant pairs.

**Interventions:**

Collection of breast milk and infants’ saliva 1 month postpartum for analysis of glucocorticoids (GCs; ie, cortisol and cortisone) using liquid chromatography– tandem mass spectrometry.

**Main Outcome Measure:**

GC rhythm in infants’ saliva and associations with vulnerability for maternal psychological distress (increased Hospital Anxiety and Depression Scale [HADS] score) or consultation at the Psychiatric Obstetric Pediatric (POP clinic), season at sampling, sex, and breast milk GC rhythmicity analyzed with SigmaPlot 14.0 software (Systat Software, San Jose, CA, USA) and regression analyses.

**Results:**

A significant biphasic GC rhythm was detected in infants, with mean peaks [standard error of the mean, SEM] at 6:53 am [1:01] and 18:36 pm [1:49] for cortisol, and at 8:50 am [1:11] and 19:57 pm [1:13] for cortisone. HADS score, POP consultation, season at sampling, and sex were not associated with the infants’ GC rhythm. Breast milk cortisol maximum was positively associated with infants’ cortisol area-under-the-curve (AUC) increase and maximum. Higher breast milk cortisone AUC increase, AUC ground, and maximum were associated with an earlier maximum in infants. Breast milk and infant GC concentrations were associated between 6:00 am and 9:00 am.

**Conclusions:**

A biphasic GC rhythm, peaking in the morning and evening, was seen in 1-month-old infants at a group level. Breast milk GC parameters might be associated with the infants’ GC rhythm, possibly caused by a signaling effect of breast milk GCs, or as an associative effect of increased mother-infant synchrony. These results contribute to an increased understanding of early life HPA-axis development.

In adults, the hypothalamus pituitary adrenal (HPA) axis displays a diurnal rhythm, peaking in the morning and with a nadir at night. However, it is not exactly clear when this adult-type rhythm is established in children, with studies reporting ages ranging from 2 weeks to 9 months in healthy infants (1–9).

A rhythm in HPA-axis activity might already be present in the human fetus. Term neonates born in the afternoon through elective cesarean section appeared to have increased cortisol concentrations compared to neonates born during other times throughout the day (10). Additionally, maternal estriol levels, partly reflecting dehydroandrostenedione production by the fetal zone of the adrenal cortex, display a 24-hour rhythm during pregnancy inversely related to maternal cortisol levels (11). Furthermore, several studies have shown data suggesting that a diurnal glucocorticoid (GC) rhythm is present from birth onward. Iwata et al. (2013) (12) described a diurnal cortisol rhythm peaking in the afternoon in newborns 2 to 11 days postpartum, while Spangler (1991) (8) found a biphasic pattern in neonates 2 to 7 days postpartum.

It is conceivable that an HPA-axis rhythm emerges prenatally and continues to develop into an adult-type rhythm after birth, with a shift from a peak in the afternoon toward a morning peak. Currently, it is not clear which factors drive the development of an adult-type diurnal rhythm of the HPA-axis. Diurnal rhythms in general are mostly regulated by the suprachiasmatic nuclei (SCN), located in the anterior hypothalamus (13, 14), and its entrainment is predominantly dependent on exogenous time cues (15). Indeed, light-dark cycles are an important regulator of SCN rhythmicity (16) and have also been shown to influence infants’ activity levels (17). Moreover, maternal depressive disorders prior to or during pregnancy were associated with sleeping problems in infants (18), while in adults, psychopathology has been linked to changes in HPA-axis activity (19, 20). A twin study has previously concluded that environmental factors outweigh the genetic contribution to the development of an HPA-axis rhythm (9). However, which exogenous factors influence the development of an HPA-axis rhythm has not been studied yet.

Maternal activity has been associated with infant activity independent of exposure to light (21), while formula milk with day/night nutrient levels in synchrony with the environment appeared to affect sleep patterns in infants (22). Breastfeeding mothers have been shown to exhibit more touching and gazing behavior toward their infants, suggestive of more interactive behavior (23), and these associations appear to be partly influenced by infant sex (24). Breast milk as well as breastfeeding itself might, therefore, also act as a possible contributor to the development of an HPA-axis rhythm. Additionally, breast milk itself contains components that might aid in the development of an adult-type GC rhythm. For example, melatonin exhibits a strong diurnal pattern in breast milk (25). Similarly, our research group has previously shown that a diurnal rhythm of cortisol and cortisone is present in breast milk, mirroring maternal HPA-axis activity (26). In rats, GCs were able to cross the intestinal epithelial barrier (27), and earlier research in humans has shown that serum cortisol levels were 40% higher in infants who were breastfed (28). Moreover, cortisol levels in maternal and infant saliva were significantly correlated in breastfed infants but not in formula-fed infants (29). Accordingly, GCs in breast milk might influence the process of HPA-axis rhythm development.

We performed an exploratory study, aimed at assessing how some exogenous factors are associated with GC rhythmicity in infants, with a focus on the association with GC rhythmicity in breast milk. Mothers with a medical history of psychopathology were oversampled in an attempt to increase the range of maternal HPA-axis activity since depression and anxiety have previously been shown to impact the GC circadian rhythm (19, 20). GC levels in the infants and breast milk were sampled at one month postpartum since HPA-axis development into an adult-type rhythm appears to still be in progress at that timepoint, while intrauterine influences are likely to have disappeared. Both cortisol and cortisone were determined since cortisone levels are higher compared to cortisol levels in both saliva and breast milk, probably due to local conversion by 11β-hydroxysteroid dehydrogenase type 2 (11β-HSD2) (30) and are, therefore, less likely to have a concentration below the lower limit of detection. Furthermore, cortisone seems to be a more reliable biomarker compared to cortisol, at least in saliva and hair (31, 32). GCs in breast milk, season at time of sampling, maternal psychopathology, and infant sex were explored as possible influencing factors, using specialized rhythm analysis software.

## Methods

### Study population

Between March 2016 and July 2017, mother-infant pairs were included from the general hospital Onze Lieve Vrouwe Gasthuis (OLVG) as well as the academic Amsterdam UMC, location VU University Medical Center (VUMC), both located in Amsterdam, as part of the Cortisol in Mother’s Milk study. The primary aim of the Cortisol in Mother’s Milk study was to research the associations between breast milk GC rhythmicity and the infant’s own HPA-axis activity, behavior, and body composition. Women included at the OLVG were recruited at the Psychiatric Obstetric Pediatric (POP) outpatient clinic where they were monitored because of an increased risk for psychopathologic complaints. Inclusion criteria were (1) infants born at term age (37–42 weeks), (2) infants with a normal birth weight (–2 to +2 SD), and (3) the mother’s intention to exclusively breastfeed for ≥3 months. Mother-infant pairs were excluded due to (1) major congenital anomalies; (2) multiple pregnancy; (3) pre-eclampsia or hemolysis, elevated liver enzymes, and low platelet count syndrome; (4) maternal alcohol consumption of >7 IU/week; and/or (5) a fever (temperature >38.5°C) at time of GC sampling. Additionally, mothers were also excluded if they used medication other than over-the-counter drugs, except for antidepressant use in the mother-infants pairs included at the OLVG.

Approval of the Medical Ethics Committee of the VUMC was obtained (protocol number 2015.524), and written informed consent was obtained from all participating mothers.

### Data collection

#### Peripartum. 

Shortly after inclusion, within the first week postpartum, mothers filled in a questionnaire pertaining to their pregnancy and birth, as well as anthropometric and demographic data.

#### One month postpartum. 

One month postpartum (±5 days), mothers collected a portion of breast milk (1–2 mL) before every feeding moment during a 24-hour period, with the use of a breast pump or via manual expression. In order to minimize intra-individual differences, we requested mothers to use the same method for all their samples. Simultaneously, before feeding, the mothers also collected their infants’ saliva, using a SalivaBio Infant’s Swab (exclusively from Salimetrics, State College, PA).

Milk and saliva were stored in the mother’s freezer, and subsequently in the laboratory at –20°C for less than 3 months prior to analysis.

At time of sampling, mothers were also asked to fill in the Hospital Anxiety and Depression Scale (HADS) questionnaire, which assessed self-reported levels of depression and anxiety symptoms (33, 34). It contained 14 questions, with 7 questions concerning depressive symptoms (hospital depression subscale; HDS) and 7 anxiety symptoms (hospital anxiety subscale; HAS). Items are scored 0 to 3, and a score of ≥8 on 1 of the 2 subscales (HDS/HAS) is indicative of clinically relevant depression and/or anxiety symptoms.

#### Laboratory. 

Total cortisol and cortisone concentrations in breast milk were determined by isotope dilution liquid chromatography–tandem mass spectrometry (LC–MS/MS) as previously published (35). In short, hexane washing was done three times to remove lipids, after adding internal standards (13C3-labeled cortisol and 13C3-labeled cortisone). Then, samples were extracted using Isolute plates (Biotage, Uppsala, Sweden) and analyzed by LC–MS/MS (Acquity with Quattro Premier XE, Milford, MA, USA, Waters Corporation). The intra-assay coefficients of variation were 4% and 5% for cortisol levels of 7 and 23 nmol/L, and 5% for cortisone levels of 8 and 33 nmol/L for LC–MS/MS measurements, respectively, while the inter-assay coefficient of variation was <9%, and the lower limit of quantitation was 0.5 nmol/L for both cortisol and cortisone. Cortisol and cortisone concentrations in saliva were determined with the same method as that for breast milk but without the hexane-washing procedure.

### Statistics

#### Regression analyses. 

 In total, 63 mother-infant pairs were included, of whom 55 pairs had valid GC levels for both mother and infant. Due to extremely high GC levels, one infant was excluded for cortisol analyses (n = 54), and another infant was excluded for cortisone analyses (n = 54).

GC levels were visualized by calculating the mean (95% confidence interval) in 2-hour time windows. Additionally, linear mixed models (LMMs), which allow correcting for intra-individual measurements, were used to determine the slope of the increasing (ie, 00:00 am – 7:00 am) and decreasing (ie, 7:01 am – 23:59 pm) part of the diurnal rhythm, in line with our previous study (26).

Next, linear regression analyses were performed, for which 7 additional mother-infant pairs were excluded because total sampling time was <8 hours and/or no samples were collected between 5:00 am and 10:00 am (ie, sample collection around the morning peak) because this could interfere with the interpretation of the rhythm parameters. This resulted in 47 included mother-infant pairs.

Infant saliva and breast milk cortisol and cortisone data were converted into rhythm parameters, which, when taken into account together, will allow a full overview of HPA-axis rhythmicity (36):

1. Area Under the Curve (AUC) with respect to the ground (AUCg) as well as AUC increase (AUCi) were calculated by using the trapezoid rule as described by Pruessner et al. (2003) (37). AUC calculations were corrected for the total sampling time since this differed between mothers. AUCg provides information on total GC exposure over the sampling time, while the AUCi is a measure of GC variability.
2. Maximum concentration measured, as a proxy for peak concentrations.
3. Time at which maximum concentration was measured, as a proxy for time of peak.

Associations between infant saliva rhythm parameters and increased HDS/HAS score, consultation at the POP outpatient clinic (as a proxy for vulnerability for maternal psychological distress), season at time of sampling (divided into two 4-month windows: 4/21 to 8/21 [summer] and 10/21 to 2/21[winter]; used as a parameter for light–dark exposure), and sex were analyzed. Season at time of sampling analyses were repeated with time windows of 3 and 6 months, as well as by determining season at birth, divided into 3-, 4-, and 6-month windows.

Additionally, the associations between breast milk and infant saliva rhythm parameters were determined.

Lastly, the associations between maternal and infant raw GC levels, split up in 3-hour time intervals, were analyzed (n = 54) by using LMMs.

Interactions with POP clinic attendance were tested. No effect modification was found, and the data were not stratified. Instead, as sensitivity analyses, analyses assessing the possible influencing factors were repeated while only including mothers who did not attend the POP clinic (n = 40).

### SigmaPlot analyses

Daily rhythmicity of cortisone and cortisol in the infants’ saliva were assessed using Gaussian peak regression with SigmaPlot 14.0 software (Systat Software, San Jose, CA, USA). The data were best fitted (ie, most optimal *P* value and adjusted R-squared, least residuals, and dependent on the least amount of variables) to the following regression formula after testing single-, double-, and triple-peak formulas (38): y = a1*exp(–.5*((x–x1)/b1)^2) + a2*exp(–.5* ((x–x2)/b2)^2), where a1 and a2 represent the estimates for the first and second peak heights, respectively; b1 and b2 represent the estimates for the full width at half maximum of the first and second peak, respectively (ie, a measure of the broadness of the peak); and x1 and x2 represent the estimates for the location (ie, the timing along the 24-hour cycle) of the first peak and second peak, respectively. Intra-individual values were taken into account and grouped together through using the shared parameters function for the regressions.

GC rhythmicity was assessed separately for the following possible influencing factors: HADS score (HDS and/or HAS <8 or ≥8), POP clinic consultation (yes/no), season at sampling (4/21 to 8/21 and 10/21 to 2/21), sex (male/female), breast milk AUCi (<p50 or >p50), breast milk AUCg (<p50 or >p50). Subsequently, t-tests were used to calculate *P* values for differences in the timing of peaks (x1 and x2). The estimates for *a* and *b* were not compared since results were often found to be unreliable, in contrast to the x-estimate.

## Results

### Population description

Table 1 shows the population characteristics. Increased HDS and/or HAS scores were found in 10 mothers, half of whom were included at the Amsterdam UMC, location VUMC. Fifteen mothers were included at the POP clinic, of whom 33.3% had clinically relevant depression (HDS) and/or anxiety (HAS) symptoms.

**Table 1.** Perinatal and Maternal Characteristics of the Study Population

	n (%) or mean ± SD
	n = 55
Gestational age (weeks)	39.7 ± 1.3
Birth weight	
grams	3550 ± 467
SDS	0.2 ± 0.9
Male sex	35 (63.6)
HAS/HDS >8	10 (18.2)
Amsterdam UMC	5 (12.5)
OLVG hospitals, POP clinic	5 (33.3)
Consulted POP outpatient clinic	15 (27.3)
Season of birth	
between 4/21 and 8/21	17 (30.9)
between 10/21 and 2/21	14 (25.5)

HAS, hospital anxiety subscale; HDS, hospital depression subscale; OLVG, Onze Lieve Vrouwe Gasthuis; POP, psychiatric obstetric pediatric; SDS, standard deviation score; UMC, University Medical Center.

### Infant GC rhythm

#### Regression analyses.

**Figure 1.**
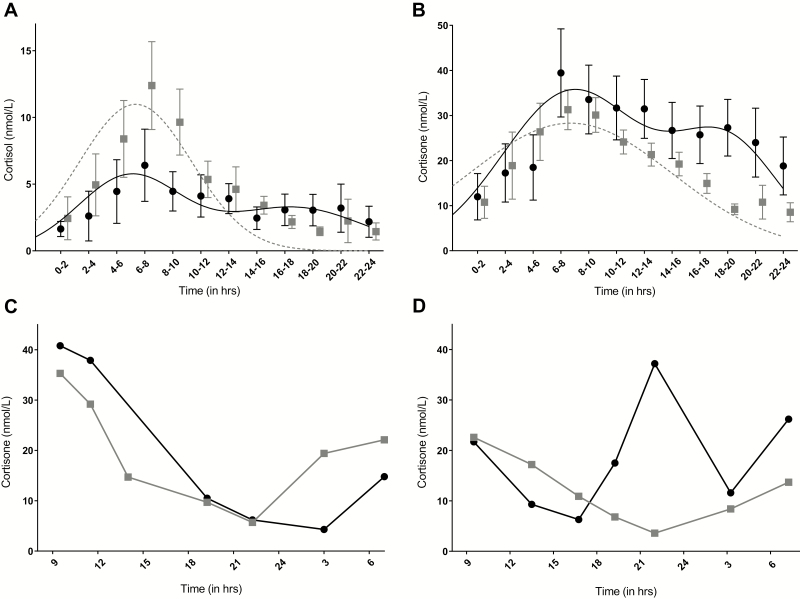
Cortisol (A) and cortisone (B, C, and D) rhythms at a group level (A and B) and for 2 individuals (C and D), in infant’s saliva (●) and breast milk (■). The formula for cortisol (A) and cortisone (B) rhythms in infant’s saliva as calculated by SigmaPlot is plotted as a continuous black line; the rhythm in breast milk is plotted as a dotted gray line.

Figures 1A and 1B show the infants’ and breast milk cortisol and cortisone levels over the day. A clear diurnal rhythm can be distinguished for both infant salivary and breast milk GC levels. LMM analyses revealed that infant salivary as well as breast milk GC levels significantly increased between 00:00 am and 7:00 am and significantly decreased between 7:01 am and 23:59 pm (all *P* values < 0.003).

#### SigmaPlot analyses.

 A significant biphasic cortisol and cortisone rhythm could be detected in the infants’ saliva. The *P* values for overall fit as well as the placement of the peaks were *P *< 0.0001 for both cortisol and cortisone. For cortisol, the first peak [SEM] occurred at 6:53 am [1:01] and the second peak at 18:36 pm [1:49]. For cortisone, the first peak occurred at 8:50 am [1:11] and the second peak at 19:57 pm [1:13]. Analyses at an individual level could not be performed due to data constraints. It could therefore not be ruled out whether results represent a true biphasic rhythm in infants or 2 separate groups of infants with a single morning or evening peak. Figures 1C and 1D show the cortisone rhythm for 2 individual infants, one with an adult-type rhythm (1C), and the other with a clear biphasic rhythm (1D).

### Rhythm influencing factors

#### Regression analyses. 

Table 2 shows the associations between rhythm parameters and possible influencing factors. Male sex was associated with a lower salivary cortisol AUCi and a lower salivary maximum cortisol concentration. However, these associations were not present when salivary cortisone rhythm parameters were analyzed. No associations were found between rhythm parameters and other factors. Repeating season at time of sampling analyses with the other time windows did not reveal any associations either [data not shown].

**Table 2.** Associations Between GC Rhythm Parameters in the Infants’ Saliva and Possible Influencing Factors

			AUCi		AUCg		Maximum		Time of Maximum	
		n	β (95% CI)	*P*	β (95% CI)	*P*	β (95% CI)	*P*	β (95% CI)	*P*
		Infants’ Saliva								
Cortisol	Increased HADS score	47	0.3 (–1.1 to 1.6)	0.68	0.1 (–1.5 to 1.7)	0.90	1.9 (–3.3 to 7.1)	0.47	–1.1 (–5.3 to 3.0)	0.59
	POP clinic consultation	46	–0.7 (–1.9 to 0.6)	0.28	–0.7 (–2.2 to 0.7)	0.31	–1.0 (–5.7 to 3.7)	0.67	–0.3 (–4.1 to 3.5)	0.88
	Season at sampling	29	–0.2 (–1.6 to 1.2)	0.75	-0.4 (–2.1 to 1.3)	0.62	–1.6 (–6.8 to 3.5)	0.52	–2.8 (–7.2 to 1.5)	0.19
	Male sex	47	–1.2 (–2.2 to –0.2)	0.02	–1.1 (–2.3 to 0.1)	0.07	–6.4 (–10.0 to -2.9)	0.001	–0.4 (–3.6 to 2.9)	0.83
Cortisone	Increased HADS score	47	4.9 (–0.7 to 10.5)	0.08	3.7 (–3.8 to 11.2)	0.32	3.5 (–12.8 to 19.9)	0.67	–0.2 (–4.1 to 3.8)	0.94
	POP clinic consultation	46	–1.4 (–6.7 to 3.9)	0.59	–2.0 (–9.0 to 5.0)	0.57	–12.3 (–26.8 to 2.2)	0.09	–2.4 (–6.0 to 1.2)	0.19
	Season at sampling	30	–4.5 (–10.0 to 1.0)	0.11	–5.5 (–12.8 to 1.9)	0.14	–8.8 (–26.1 to 8.4)	0.30	–0.3 (–4.3 to 3.7)	0.88
	Male sex	47	–0.9 (–5.4 to 3.6)	0.68	–0.9 (–6.9 to 5.0)	0.76	–5.5 (–18.1 to 7.1)	0.38	2.1 (–1.0 to 5.1)	0.18

Values represent β (95% CI) as analyzed with linear regression.

Increased HADS score: ≥8 on the HDS and/or HAS subscore.

Season at sampling was divided into 4-month windows: 21/4 to 21/8 (summer) and 21/10 to 21/2 (winter).

Abbreviations: AUCg, area under the curve ground; AUCi, area under the curve increase; CI, confidence interval; GC, glucocorticoid; HADS, Hospital Anxiety and Depression Scale; HAS, hospital anxiety subscale; HDS, hospital depression subscale; POP, psychiatric obstetric pediatric.

Breast milk maximum cortisol levels were positively associated with salivary cortisol AUCi and maximum levels in the infant (Table 3). Additionally, higher breast milk cortisone AUCi, AUCg, and maximum concentrations were associated with an earlier time of salivary maximum cortisone in the infant. No other associations were found.

**Table 3.** Associations Between GC Rhythm Parameters in the Infants’ Saliva and Breast Milk

			Infants’ Saliva							
			AUCi		AUCg		Maximum		Time of Maximum	
			β (95% CI)	*P*	β (95% CI)	*P*	β (95% CI)	*P*	β (95% CI)	*P*
Breast Milk	Cortisol	AUCi	0.2 (–0.04 to 0.4)	0.10	0.2 (–0.1 to 0.5)	0.19	0.7 (–0.2 to 1.6)	0.13	–0.4 (–1.1 to 0.3)	0.26
		AUCg	0.2 (–0.02 to 0.4)	0.08	0.2 (–0.1 to 0.4)	0.16	0.6 (–0.1 to 1.4)	0.11	–0.4 (–1.0 to 0.3)	0.27
		Maximum	0.1 (0.01 to 0.12)	0.02	0.1 (–0.01 to 0.1)	0.12	0.2 (0.02 to 0.4)	0.03	–0.2 (–0.3 to 0.02)	0.07
		Time of maximum	0.0 (–0.2 to 0.2)	0.90	0.1 (–0.1 to 0.4)	0.27	–0.2 (–1.0 to 0.5)	0.57	0.4 (–0.3 to 1.0)	0.25
	Cortisone	AUCi	0.4 (–0.2 to 1.0)	0.18	0.3 (–0.4 to 1.1)	0.41	1.5 (–0.1 to 3.1)	0.07	–0.4 (–0.8 to –0.1)	0.03
		AUCg	0.1 (–0.3 to 0.5)	0.66	0.1 (–0.5 to 0.7)	0.76	0.9 (–0.3 to 2.1)	0.13	–0.3 (–0.6 to -0.1)	0.02
		Maximum	0.2 (–0.1 to 0.4)	0.18	0.0 (–0.3 to 0.3)	0.91	0.4 (–0.2 to 1.1)	0.20	–0.2 (–0.4 to –0.04)	0.01
		Time of maximum	0.2 (–0.7 to 1.2)	0.65	0.8 (–0.4 to 2.1)	0.20	0.7 (–2.1 to 3.4)	0.63	0.4 (–0.3 to 1.0)	0.28

Values represent β (95% CI) as analyzed with linear regression.

Abbreviations: AUCi: area under the curve increase, representing GC variability;

Table 4 shows the associations between raw data of breast milk and infant salivary GC concentrations, divided into 3-hour time intervals. A positive association was found for both cortisol and cortisone between 6:00 am and 9:00 am, while no associations were found in the other time windows. Repeated analyses with natural log-transformed GC levels found similar results [data not shown].

**Table 4.** Associations Between Breast Milk and Infants’ Saliva GC Concentrations per 3-Hour Time Interval

	Cortisol		Cortisone	
Time Interval (n)	β (95%CI)	*P*	β (95%CI)	*P*
0:00 am–3:00 am(20/21)	–0.1 (–0.4 to 0.1)	0.35	–0.3 (–0.9 to 0.2)	0.21
3:00 am–6:00 am (23)	0.2 (–0.1 to 0.5)	0.23	0.3 (–0.1 to 0.7)	0.14
6:00 am–9:00 am (42)	0.2 (0.03 to 0.5)	0.03	0.7 (0.1 to 1.3)	0.03
9:00 am–12:00 pm (54/52)	0.0 (–0.3 to 0.3)	0.96	0.0 (–0.6 to 0.6)	0.94
12:00 pm–15:00 pm (44)	0.1 (–0.04 to 0.3)	0.11	0.6 (–0.1 to 1.3)	0.09
15:00 pm–18:00 pm (43)	–0.1 (–0.7 to 0.5)	0.78	–0.1 (–0.8 to 0.7)	0.88
18:00 pm–21:00 pm (39)	0.1 (–0.7 to 1.0)	0.78	–0.2 (–1.4 to 1.0)	0.69
21:00 pm–24:00 pm (35)	–0.3 (–1.4 to 0.7)	0.51	–0.3 (–1.6 to 1.0)	0.63

Values represent β (95% CI) as analyzed with linear mixed models, while adjusting for intra-individual measurements.

Abbreviations: CI, confidence interval.

When repeating the analyses while excluding the mother-infant pairs who attended the POP clinic (n = 15), small changes were found: 2 associations became significant, whereas 3 associations became nonsignificant (38). Additionally, the association between breast milk and infant GC concentrations collected between 6:00 am and 9:00 am also disappeared (online Supplementary Table 4 (38)).

#### SigmaPlot analyses. 

Table 5 shows the mean differences in time of peak for the studied possible influencing factors. Infants of mothers who attended the POP clinic had a significantly earlier time of the second salivary cortisol peak. Time of the first salivary cortisol peak was earlier in infants with a breast milk AUCi and AUCg > p50, and time of the second salivary cortisol peak was significantly earlier in infants with a breast milk AUCg > p50. No differences were found in the timing of the salivary cortisone peaks.

**Table 5.** Mean Differences (in Hours) in Time of Peak for Possible Influencing Factors

	Cortisol		Cortisone	
Influencing Factors	Peak 1	Peak 2	Peak 1	Peak 2
HADS score	0:18 ± 2:00	6:30 ± 3:48	-0:54 ± 6:06	0:36 ± 6:36
POP clinic consultation	-1:36 ± 1:12	-5:12 ± 1:42^**^	-1:42 ± 2:12	-2:00 ± 2:18
Season at sampling	-2:06 ± 1:24	-2:30 ± 2:48	-1:54 ± 4:12	-2:12 ± 3:42
Sex	-0:12 ± 2:42	1:00 ± 3:06	-1:54 ± 3:12	-0:24 ± 3:18
AUCi breast milk	-3:12 ± 1:30^*^	-3:12 ± 3:18	-1:00 ± 4:42	-0:18 ± 5:18
AUCg breast milk	-2:24 ± 0:54^*^	-6:00 ± 1:42^***^	-1:18 ± 3:30	0:18 ± 3:42

Values represent mean differences ± SEM in hours as tested with t-tests.

*P value < 0.05; **P value < 0.01; ***P value < 0.001.

HADS score was dichotomized as <8 or ≥8 on the anxiety and/or depression subscore.

Season at sampling was divided into 4-month windows: 4/21 to 8/21 (summer) and 10/21 to 2/21 (winter).

AUCi and AUCg of breast milk were dichotomized as <p50 and > p50.

Abbreviations: AUCg, area under the curve ground; AUCi, area under the curve increase; HADS, Hospital Anxiety and Depression Scale; SEM, standard error of the mean.

When repeating the analyses while excluding mother-infant pairs who attended the POP clinic (n = 15), only one association became significant (38). None of the other associations changed.

## Discussion

In this study, we have shown that full-term infants at a group level have a biphasic diurnal GC rhythm at the age of 1 month with peaks in the morning as well as in the evening. Increased risk for maternal psychopathologic complaints (increased HADS score or POP clinic consultation), season at sampling, and sex were not associated with the infants’ cortisol and cortisone rhythm parameters. Maternal GCs in breast milk might be associated with the infants’ GC rhythm since a more variable breast milk GC rhythm appears to be associated with an earlier time of maximum in infants. However, the sample size of the study was small, and results were not consistent between cortisol and cortisone parameters and should therefore be interpreted with caution.

The most striking finding of our study is the double peak that was found in the infant GC rhythm at a group level. A double peak has been described before (8, 39, 40), although those peaks were not related to a specific time of day. Several explanations are possible for the presence of this double-peak rhythm. First, the double peak was seen at a group level. Since analyses at the individual level could not be performed, it is possible that the biphasic rhythm was caused by 2 or more separate groups of infants, with some peaking in the morning, while others had a peak occurring in the evening. However, visualizing the data per mother-infant pair revealed that several infants appeared to have a double peak, with examples shown in Figures 1C and 1D. Alternatively, a double peak could be a part of the development toward an adult-type GC rhythm. Several studies have shown that an adrenal rhythm, with a peak in the afternoon/evening, might be present in utero (10, 11). After birth, under the influence of exogenous factors (15), an adult-type adrenal rhythm develops. The fetal GC peak in the evening could slowly disappear, and a morning peak might take its place. During this development, a transitional period might exist in which the remnants of the fetal evening peak and the beginnings of an adult-type morning peak are both present. However, to test this hypothesis, longitudinal data are necessary.

Nevertheless, as far as we are aware, this is the first study to show the presence of a double peak at the age of 1 month, although a biphasic rhythm has been reported at a younger age (8). Ivars et al. (2015) have previously shown the presence of a significant GC rhythm at this age (2) but did not report a double peak. Other studies have reported a later establishment of a GC rhythm in infants (1, 7, 40, 41). These differences in outcomes could be due to heterogeneity in statistical methods. Price et al. (1983) (6) defined a circadian rhythm as a higher value in the morning than in the evening, with a steady decline throughout the day. Santiago et al. (1996) (7) and Antonini et al. (2000) (41) considered a circadian rhythm to be present when afternoon and evening values were 83.5% or less of the morning concentration, whereas Ivars et al. (2015) (2) used a ratio of <0.8 between morning and evening levels to determine the presence of a rhythm. De Weerth et al. (2003) (1) used hierarchical linear modeling. All of these methods are based on the premise that a rhythm is present when there is a linear decline in GC concentrations. However, as we have shown, at a group level a second peak is present in the evening. Since this peak is on average lower than the morning peak, it is possible that a circadian rhythm is considered to be present according to these other methods, while the second peak is overlooked.

The possible influencing factors considered in this study were not significantly associated with the salivary infant GC rhythm, although when analyzing only mother-infant pairs who did not attend the POP clinic, some effects of season of sampling were found on the timing of the cortisol, but not cortisone, peak of the infants. However, several associations were found between breast milk and salivary infant GC parameters. The cortisol maximum in breast milk was associated with more salivary cortisol variability, a higher maximum, and earlier time of maximum in infants according to linear regression analyses, and higher breast milk cortisol variability and total exposure were associated with earlier times of salivary cortisol peaks as analyzed by SigmaPlot. More cortisone variability, total exposure, and a higher maximum in breast milk were associated with an earlier salivary cortisone peak in the infants. Additionally, breast milk and infant GC concentrations were correlated between 6:00 am and 9:00 am (ie, during the morning peak). Whether these findings are due to a true association is unclear since findings were not consistent between cortisol and cortisone parameters. Cortisol concentrations especially are difficult to interpret in the saliva samples of infants since 26% of the valid measurements were below the lower limit of detection (1 nmol/L). Cortisone levels were higher and did not reach the lower limit of detection, probably due to local conversion by 11β-HSD2 (30). Additionally, cortisone has been found to be more reliable than cortisol, at least in saliva and hair (31, 32). The results, which use cortisone parameters, are therefore likely to be more trustworthy. However, inert breast milk cortisone would have to be converted to active cortisol in the infant. We have previously speculated that the gut microbiota might play a role in this (42). Additionally, 11β-HSD1 is expressed in the human intestine, liver, and in the SCN (43–45). Even if only the analyses performed with cortisone parameters are reliable, it would seem that a more variable cortisone rhythm with a high peak in breast milk could bring the time of the morning peak forward in infants, although these associations were only found in regression analyses. In light of our previous hypothesis, this could mean that GCs in breast milk might aid in the transition from a fetal to an adult-type GC rhythm. The morning peak of GCs in breast milk could have a role in this transition, since breast milk and infant salivary GC levels were significantly correlated during this time interval. The effects of breast milk GCs could be caused by directly influencing GC concentrations in infant serum, although this is less likely due to the low absolute concentrations, or by acting as a signaling function in the infant’s intestines (ie, the gut-brain axis hypothesis) (46, 47). Alternatively, the associations might not be due to causality but because another factor influences the maternal and infant HPA-axis in a similar fashion. Breastfeeding is associated with more responsive parenting (48) and increased maternal sensitivity (49) compared to formula feeding. Increased mother-infant synchrony caused by breastfeeding might therefore be a factor itself in influencing both maternal and infant GC rhythms.

This study has several strengths and limitations. First, our study’s design enabled us to collect breast milk and saliva samples at all hours of the day, with a total of 967 GC samples from 55 mother-infant pairs. Detailed analyses of infant and breast milk rhythm could therefore be performed. Second, our analytical approach allowed for a detailed overview of infant GC rhythmicity, revealing a double peak. Third, maternal distress was measured at time of sampling, and its associations with the infants’ HPA-axis activity could be taken into account. Our study also has its limitations. Due to collection errors, some infant samples did not contain enough saliva for laboratory analyses. This meant that several mother-infant pairs had to be excluded because no valid samples were available around the time of the expected (maternal) peak (ie, 5:00 am – 10:00 am) or because total sampling time was not sufficient (ie, <8 hours). However, these exclusion criteria attempted to reduce the chances of a bias since GC levels of the excluded mother-infants pairs were likely to have lower maximum concentrations as well as AUCs. Additionally, our sample size of 55 mother-infant pairs is quite limited, and the number of subjects that attended the POP clinic (n = 15, 27.3%) and/or had an increased HADS score (n = 10, 18.2%) was also small, although the incidence of increased psychological distress in this study was comparable with the prevalence in the general population (50). The statistical power therefore might have been too low to detect certain associations. It also required us to pool the available data, and individual as well as adjusted analyses were not possible. On the other hand, with the exception of 1 study (2), our sample size was bigger than other studies assessing HPA-axis development in early life in term infants (1, 3, 6–8, 40, 41). Moreover, although we studied several possible influencing factors, we did not take all determinants into consideration. For instance, no data were collected about timing of daytime naps, while sleep has previously been associated with GC levels in infants (1, 8, 51, 52). In the previous studies, most of these associations were found in older infants than the ones included in this study, but an effect cannot be ruled out. However, daytime naps were associated with decreased cortisol levels immediately after the nap (52), and they are unlikely to explain the biphasic rhythm found in this study. Sleeping through the night was found to be associated with a more pronounced circadian rhythm (1), but it has previously also been shown that the establishment of a diurnal GC rhythm precedes a sleep-wake rhythm (3). Additionally, the lack of a formula-fed control group limited our possibilities with regard to testing the associations between breastfeeding and GC rhythmicity in the infants, and the effect of the act of breastfeeding as a whole could not be studied. Furthermore, the possibility of a selection bias cannot be excluded because stressed mothers with infants who slept restlessly (indicative of a lack of rhythm) were probably less likely to participate, and it is likely that the study population does not reflect the general or the POP clinic population. However, we did not aim to find reference ranges but designed this study to analyze effects of inter-individual variation in breast milk rhythmicity. We did not collect data on mothers who were eligible for inclusion but opted out of participating, and a selection bias could consequently not be tested. Additionally, mothers who attended the POP clinic might have had other reasons for not participating compared with mothers who did not attend the POP clinic, which could have further skewed results. Lastly, a longitudinal study design would have enabled us a better understanding of HPA-axis development and which factors are of influence. However, we aimed to make this study as noninvasive as possible, and therefore decided to have mothers collect milk and saliva samples during 1 day only.

In conclusion, a biphasic GC rhythm appears to be present at a group level at the age of 1 month, with a peak in both the morning and the evening, which might be part of the developmental process toward an adult-type GC rhythm. Increased risk for maternal psychopathologic complaints (increased HADS score or POP clinic consultation), season at sampling, and sex were not associated with infant GC rhythmicity in this study. However, breast milk GC parameters might be associated with the infants’ GC rhythm, which might be due to a causal signaling effect of breast milk GCs or because of an associative effect due to increased mother-infant synchrony. Although future studies should further elucidate HPA-axis development in early life, preferably with a longitudinal design and including a formula-fed control group, this exploratory study contributes to an increased understanding of this process, especially with regard to the role of breast milk.

## Abbreviations

11β-HSD: 11β-hydroxysteroid dehydrogenase
AUCi: area under the curve increase
AUCg: area under the curve ground
GC: glucocorticoid
HADS: Hospital Anxiety and Depression Scale
HAS: hospital anxiety subscale
HDS: hospital depression subscale
HPA: hypothalamus-pituitary-adrenal
LMM: linear mixed model
OLVG: Onze Lieve Vrouwe Gasthuis
POP: psychiatric obstetric pediatric
SEM: standard error of the mean
SCN: suprachiasmatic nuclei
UMC: University Medical Center
VUM: VU University Medical Center

## Additional Information

***Disclosure Summary*:** The authors have nothing to disclose.

Data Availability: The datasets generated during and/or analyzed during the current study are not available but are available from the corresponding author on reasonable request.
